# Seeing past the tip of your own nose? How outward and self-centred orientations could contribute to closing the green gap despite helplessness

**DOI:** 10.1186/s40359-023-01128-z

**Published:** 2023-03-24

**Authors:** Magdalena Adamus, Jakub Šrol, Vladimíra Čavojová, Eva Ballová Mikušková

**Affiliations:** 1 Centre of Social and Psychological Sciences, Slovak Academy of Sciences, Dúbravská Cesta 9, 841 04 Bratislava, Slovakia; 2 Faculty of Economics and Administration, Masaryk University, Lipová 41a, 602 00 Brno-střed, Czech Republic

**Keywords:** Environmental concern, Pro-environmental behaviour, Green gap, Collectivism-individualism, Future orientation, Prosocial tendencies, Helplessness

## Abstract

**Background:**

The present study explored moderators of the relation between environmental concerns and pro-environmental behaviour that could help close the green gap.

**Methods:**

A sample of 500 individuals (250 women) participated in the study. Apart from socio-demographic characteristics, participants answered questions about their environmental concerns and pro-environmental behaviour, collectivism and individualism, time orientation and emotional responses to climate change.

**Results:**

Our results corroborate the view that collectivism, future orientation and prosocial tendencies may form a single component of outward orientation, while individualism and immediate orientation form self-centred orientation. Generally, outwardly oriented individuals and those less self-centred reported more pro-environmental behaviour. However, strongly self-centred individuals, even when reporting elevated helplessness, showed increased involvement in pro-environmental behaviour once their concerns were high.

**Conclusions:**

The study contributes to the literature by pointing out that both outward and self-centred orientations have the potential to insulate individuals against the negative effect helplessness may have on pro-environmental behaviour. This could inform strategies that would both prompt individuals already concerned to act and arouse more concern among those who are not yet preoccupied with climate change.

**Supplementary Information:**

The online version contains supplementary material available at 10.1186/s40359-023-01128-z.

## Background

Environmental awareness has increased over the recent years and a vast majority of European citizens believe that climate change will have adverse effects on their lives [1]. The awareness is related to environmental concerns and the belief that embracing more sustainable everyday behaviours is becoming a necessity in order to prevent or at least mitigate negative anthropogenic environmental impacts. Yet, despite growing concerns, studies repeatedly show weak correlations between concerns and pro-environmental behaviour (PEB)—many more people seem to be concerned with climate change than actually are involved in actions preventing it. Consequently, there remains a gap (so-called green gap) between expressed environmental concerns and adopted PEB [2, 3]. The literature identifies numerous barriers preventing people from acting in a pro-environmental vein including limited cognition, adopted ideologies and worldviews (e.g. political or religious), flawed risk perception or favourable comparisons with others who do even less [4–6]. Furthermore, the data show that people who feel helpless or only moderately self-efficient in reducing human impacts on the environment could end up being demotivated despite experiencing high environmental concerns [7].

Notwithstanding the barriers, the literature reflects a consensus that PEB involves personal commitment. A person wishing to behave pro-environmentally needs to put an additional effort to seek sustainable alternatives and cover the costs to make behavioural patterns greener [8–10]. The willingness to make this additional effort could be associated with certain individual characteristics. For instance, Unanue et al. [11] pointed out that individuals committed to extrinsic, materialistic goals (money, fame, image) are less likely to get involved in behaviours that promote environmental protection. Conversely, those who prioritise intrinsic goals (self-development, community involvement, relationships), adopt more sustainable behaviours in areas as wide as energy conservation, mobility and transportation, waste avoidance or consumerism. Therefore, transcending one's habits and immediate self-interest could provide a sense of justification for incurring the costs for the sake of future environmental benefits. Previous research supported the view that certain characteristics, such as being collectivistic, future-oriented and having prosocial motivations, could contribute to embracing more sustainable behaviour and bridging the concern-behaviour gap.

The present study investigates whether characteristics associated with transcending narrow self-interest moderate the relation between environmental concern and PEB, contributing thus to closing the green gap. The study also explores whether it is possible to consider those individual characteristics jointly and form two latent constructs and whether those constructs could explain PEB above and beyond demographic characteristics and emotional factors. Consequently, recognising that collectivism, future orientation, and prosocial motivations—usually studied separately—share a common feature of self-transcendence, the study proposes an umbrella term *outward orientation* for forms of concern that go beyond here and now and surpass selfish motivations. Outward orientation is contrasted then with *self-centred orientation* that reflects immediate, individualistic and selfish interests. The results show that people with strong either outward or self-centred orientation could behave pro-environmentally although, perhaps, driven by different motivations. More importantly, the two constructs have a potential to act as moderators in the relation between concerns and behaviour helping to close the green gap. The findings have practical consequences for encouraging people to put more effort in sustainable behaviour. People with more self-centred characteristics are not uninterested in PEB. The necessary condition for their involvement in PEB is a high level of environmental concerns. They may, thus, respond to different cues and require more tailor-made communication that matches their rather extrinsic motivations. Finally, our results indicate that the effects remain robust after controlling for helplessness—a factor that could discourage people from behaving sustainably regardless of their individual characteristics.

### Pro-environmental behaviour as transcending self-interest

The political polarisation around environmental issues creates an atmosphere in which sustainable choices are driven more by values than scientific evidence [12, 13]. For instance, the endorsement of altruistic and biospheric (as opposed to self-enhancing) values systematically shows to contribute to more pro-environmental behavioural intentions [14–19]. Apart of the Schwartz’s value orientations, the literature pinpointed other characteristics associated with transcending self-interest that could enhance PEB. Specifically, putting collective welfare ahead of personal interests (collectivism), being able to put aside immediate benefits in favour of the future (time orientation), and caring for the wellbeing of other individuals (prosocial motivations) all showed to be positively correlated with the willingness to behave pro-environmentally [3, 20, 21]. Based on the literature, the following sections discuss the role of the latter three characteristics in promoting sustainable life-style and consumption patterns. It seems likely that each of the characteristics contributes its share to the understanding that even if individuals do not suffer from immediate and direct impacts of climate change, they are not exempt from the responsibility for protecting environment as well as contemporary people and future generations.

#### Understanding associations between collectivism and individualism and pro-environmental behaviour

It is often pointed out that collectivism is more likely to be directly related to PEB than individualism. For instance, Arisal and Atalar [22] found that in Turkey more collectivistic-oriented individuals tend to express greater environmental concern and more ecological purchase intentions. Similarly, in a study comprising individuals from South Korea and the United States, Cho et al. [23] observed both horizontal and Confucian collectivists express greater intentions to buy green products and from green producers. At the country level, Komatsu et al. [24] found that more individualistic countries have higher Ecological Footprints (i.e., their negative impact on the environment is larger) and suggested that individualists have lower acceptance for anthropogenic origins of climate change. Collectivists (compared to individualists) could be more sensitive to injunctive and descriptive social norms about PEB and recognise that their dedication to common good is more valuable than achieving immediate pleasures and self-centred aims. Moreover, egalitarian individuals perceived fewer barriers preventing them from behaving pro-environmentally [25]. Thus, the sense of belonging to a group and the belief that individuals benefit from the group’s wellbeing, place collectivism among strong direct antecedents of PEB.

However, after a more thorough analysis taking the concern-behaviour gap into account, the relation appears less straightforward. For instance, Eom et al. [26] found that in individualistic cultures correlations between environmental concerns and actions were stronger than in collectivistic ones. Concurrently, Tam and Chan [3] observed that higher levels of country-level individualism and looseness help to translate concerns into PEB more smoothly. One drawback of the culture-level analysis, however, is that individualistic countries tend to be also wealthier and more developed and thus other either economic or cultural factors could confound the analysis. Indeed, in a meta-analysis involving 66 studies from 28 countries, Morren and Grinstein [27] found that both the state of (economic and human capital) development and country-level individualism come hand in hand in bridging the green gap. Recently, the results were substantiated by Zheng et al. [28] who found that, even after controlling for a series of country-level characteristics including wealth, individualism contributes to setting more ambitious goals within the Paris Agreement. Perhaps, when individualists experience high levels of environmental concerns they also more easily find motivation to act pro-environmentally and thus alleviate their worries. It seems that once individualists set environmental objectives for themselves they may also have a stronger sense of agency in pursuing them. Paradoxically, thus, it appears that while collectivism may have a considerable direct effect on the tendency to behave pro-environmentally, individualism may be key in spanning the green gap.

To delve deeper into the relation between collectivism and individualism and PEB, we turned our attention to individual-level analysis and employed a four-dimensional typology with two bipolar subscales [29]. Apart from Individualism-Collectivism, the authors added the Vertical-Horizontal continuum. The resulting typology distinguishes two forms of collectivism: vertical (VC) and horizontal (HC). Generally, collectivists tend to see themselves as submerged in a group they belong to, but they differ in their preference for a hierarchy between groups. While horizontal collectivists express egalitarian views about different groups, vertical collectivists tend to believe in intergroup rivalry. We expected to replicate previous findings of the direct relation between collectivism and PEB and further explore the moderating effects of collectivism and individualism in the concern-behaviour relation.

#### Time orientation as an antecedent of pro-environmental behaviour

Despite numerous arguments to the contrary, some still perceive the consequences of climate change as distant and although outright denialism is no longer a viable option, many stakeholders who profit from the status quo attempt to delay the change by undermining the gravity of the situation [31]. Thus, the willingness to respond to future threats requires sensitivity to consequences an individual may have neither fully understood nor experienced yet. Unlike future-oriented individuals, those who focus on immediate rewards could underestimate risks and costs of future climate disasters, fail to recognise the urgency to act now to prevent them and remain undeterred by the future consequences of their current actions [32]. They may remain unencouraged by potential environmental gains—likely cherished rather by future generations—when the costs need to be borne now. Consequently, individuals who focus on immediate rewards are believed to be less environmentally concerned and get involved in fewer PEBs. Along with individualism and collectivism, time orientation belongs to factors with best-established relation to PEB and environmental concern [33, 34]. For instance, those scoring high in future-orientation measured by the Considerations of Future Consequences scale (CFC) expressed a stronger preference for biofuels considered to be a more ecologically-conscious choice [34]. On the contrary, those who reported being more immediate-oriented preferred gasoline. Generally, people scoring high in future-orientation (measured by the CFC subscale) are more concerned with climate change and express greater willingness to adopt green behavioural patterns [33, 35]. Country-specific findings follow the individual-level patterns with citizens of more future-oriented countries being also more committed to pro-environmental goals. Their enhanced environmental concern has a tangible financial dimension as well: future-oriented people are more willing to pay (both higher taxes and prices) to mitigate global warming, indicating that they are more likely to transcend their narrow self-interest [36].

Finally, apart from cultural orientation, Tam and Chan [3] investigated the moderating effect of country-level time orientation on closing the green gap. Analysing 32 countries, the authors observed the gap to widen with the increase in present-orientation. Giving precedence to the present, it seems, hampers the effect environmental concerns may have on behaviour. The findings suggest that—while being a significant antecedent of both environmental concern and PEB—for closing the green gap, future orientation is of lesser importance. Instead, it seems key not to be presently-oriented. In line with the extant literature, thus, we expected future-oriented individuals to be more environmentally concerned and invest more effort in PEB. Building on these findings, we also explored the moderating role of time-orientation in closing the green gap.

#### Pro-environmental behaviour as a reflection of prosocial motivations

Another form of going beyond self-interest is altruism. Having altruistic motivations may incline an individual to overcome egoistic goals and focus on the public good of mitigating climate change. Conversely, self-centred, rational individuals that act in their immediate best interest, deplete the public good and jointly contribute to the deterioration of environmental conditions by being, for instance, less environmentally concerned or choosing less ecologic options [34, 37]. In the context of PEB, pure altruism is impossible since a clean environment is by and of itself a public good—i.e., a person that contributes is also entitled to reap the benefits of their contribution. Any PEB that contributes to a cleaner environment has a side effect of providing an individual with additional gains. However, apart from tangible contribution to pro-environmental efforts, an individual may find PEB (as a form of prosociality) psychologically rewarding. An important concept, in this context, is a warm glow.

Kahneman and Knetsch [21] showed that the willingness to contribute to pro-environmental causes reflects the willingness to pay for moral satisfaction. In other words, individuals valued their satisfaction more than they valued the improvement of environmental conditions. More recently, Hartman et al. [38] showed that a warm glow explains pro-environmental intentions better than altruism, indicating that PEB may be driven by impure variations of altruism that benefit individuals themselves. It seems, thus, that although altruistic traits may directly contribute to PEB, they are not necessary for embracing more PEB. Instead, a person may be willing to adopt more sustainable behaviour motivated by a positive impact the behaviour has on their psychological wellbeing or social image. In this view, environmental and societal benefits come as a welcomed addition but not a necessary condition of PEB. In this study, thus, we proposed to investigate a wider set of prosocial motivations regardless of any personal gains they may provide to an individual. We expected prosocial motivations to be positively related to environmental concerns and PEB. In the second step, we delved deeper into the question of whether prosocial motivations—as an element of outward orientation—have the potential to strengthen the relation between concerns and PEB.

### Emotion-driven pro-environmental behaviour (concern and helplessness)

Apart from the aforementioned individual characteristics that could be associated with more effective transition from concerns to actions, green gap could arise due to maladaptive responses to the experienced threat. Adaptive responses to concerns involve cognitive processes focused on problem-solving. On the other hand, unconstructive responses may lead to excessive worries but also avoidance and, as a consequence, inaction [39]. Thus, irrespective of an individual’s sensitivity to outward goals, the psychological literature stresses the importance of a belief that an individual's action has a tangible impact on achieving a desirable outcome. In other words, the belief that the action is not futile [40]. Alas, ESS data [1] show that across 23 countries people consistently do not believe they can make a meaningful difference by reducing their energy consumption. Generally, the findings about self-efficacy paint a rather grim picture. Even individuals concerned with climate change may refrain from actions, discouraged by their belief that individual efforts mean little in tackling climate change.

Given that slowing down global warming requires coordinated actions of the vast majority of the global population, concepts related to self-efficacy may provide at least partial explanation why concerned people do not behave more pro-environmentally. For instance, [3] investigated whether the external locus of control may interfere with the concern-behaviour relation. Both at the country and individual level, the analysis showed that a greater belief in external control loosened the relation between concerns and PEB serving as a barrier in embracing more sustainable behaviour. Similarly, in a study by Nguyen et al. [9], perceived consumer effectiveness turned out to be an important moderator strengthening the impact concerns may have on sustainable consumption patterns. Specifically, the more individuals believed that individual contributions make a difference and green products have a considerable economic and environmental impact, the more often they expressed intentions to embrace more PEB and preferences for buying green products. Finally, a similar pattern was observed by Landry et al. [7] about learned helplessness understood as a tendency to behave helplessly across various behavioural domains. Once individuals reported low levels of helplessness, environmental concerns predicted self-reported PEB and donations to an environmental organisation. In other words, an elevated sense of helplessness contributed to the widening of the green gap. To delve deeper into the causes of the green gap, we thus explored the moderating role of domain-specific, environmental helplessness in the relation between environmental concerns and actions.

## Materials and methods

### Participants and procedure

The final sample consisted of 500 participants (descriptive statistics is in Table 1), who were recruited and compensated by the external agency. The sample was representative of the Slovak population concerning age and gender. Post-hoc sensitivity analysis carried out in GPower (40) showed that the current sample size provided sufficient statistical power (0.80) to detect even relatively small effect sizes with correlations of *r* > 0.125 and single regression coefficients of *f*^2^ > 0.012 in a regression with 11 predictors (see moderation analyses below) with 5% error probability.

**Table 1:** Descriptive statistics of the sample

	Age		
	Men	Women	All
*N*	250	250	500
*M*	45.77	42.87	44.32
*SD*	15.33	15.88	15.66
	Education		
	Men	Women	All
Elementary/incomplete high school	14.4%	12.4%	13.4%
Complete high school education	43.2%	49.2%	46.2%
Some college or complete college	42%	37.6%	40.4%

The survey took the form of an online questionnaire created in Qualtrics. The data were collected as part of a larger study of prosocial behaviour about socially controversial topics (COVID-19, vaccination, climate change) but was intended to form a separate study. After reading and signing an informed consent form, the participants first answered several demographic questions and then proceeded to the blocks of questions related to individual differences (collectivism/individualism, consideration of future consequences, prosocial tendencies), environmental concern, helplessness related to climate change, and PEB. The whole survey took about 30 min and included four attention-check questions (e.g. In this question please press “I pay attention” on the scale below). 58 respondents who failed to answer correctly were excluded from the analysis. Descriptive statistics and internal consistency estimates for all measured variables are in Table 2. Analyses were conducted using jamovi 1.8. software [41] and SPSS Process macro by Hayes [42].

**Table 2:** Descriptive statistics for measured variables

	*M*	*SD*	α	Skewness	Kurtosis	Original psychometric propertie**s**
*Individualism/ collectivism*						(Sivadas et al. [29]; Study 5) χ^2^ = 129.95, df = 71, p < 0.000),. GFI = 0.82, AGFI = 0.73, RMSR = 0.33, CFI = 0.82, NFI = 0.69, RMSEA = 0.091
Horizontal individualism	3.51	0.79	0.63	− 0.11	− 0.28	α = 0.806
Vertical individualism	2.92	0.88	0.74	− 0.27	− 0.21	α = 0.709
Horizontal collectivism	3.58	0.73	0.72	− 0.36	0.30	α = 0.645
Vertical collectivism	3.39	0.70	0.59	− 0.59	0.62	α = 0.745
*Future orientation*						Joireman et al. [43] The final 2-factor model fit the data well, SBχ2(69) = 97.69, p =0 .013, GFI = 0.943, CFI = .0.965, RMSEA = .0.043 (LL = 0. 020, UL = 0.062)
Future consequences	4.77	1.02	0.85	− 0.19	0.53	α = 0.82
Immediate consequences	3.72	1.08	0.82	0.06	− 0.01	α = 0.84
*Prosocial behaviour*						Carlo and Randall [45]
Prosocial tendencies	3.55	0.66	0.88	− 0.23	0.47	α = 0.54–0.88
*Emotional reactions*						Kohút et al. [47], Šrol et al. [49]
Environmental concern	4.46	1.55	0.93	− 0.34	− 0.32	–
						(measured by one item)
Helplessness	4.15	1.56	0.91	− 0.31	− 0.53	α = 0.69
*Outcome variable*						
Pro-environmental behaviour	2.01	0.30	0.70	− 0.10	0.25	–
						(created for this study)

The table contains averages, standard deviations, skewness and kurtosis values, and internal consistency estimates (Cronbach’s α) for all reported variables

### Measures

Before the main part of the study, participants answered several questions related to their demographic information, political attitudes, and the importance of religion.

#### Outward orientation

*Collectivism/Individualism* was measured by the 14-item HVIC scale [29], which comprises four subscales. *Horizontal individualism* reflecting the sense of being self-reliant without a tendency to compete with others. The subscale contains three items (e.g., “I enjoy being unique and different from others in many ways”). The *vertical individualism subscale* focuses on competitively establishing one's status (e.g., “I enjoy working in situations involving competition with others”). *Horizontal collectivism* is understood as a tendency to acknowledge interdependence and value social relations and common goals. The subscale contains 4 items (e.g., “My happiness depends very much on the happiness of those around me”). Finally, the *vertical collectivism* subscale expresses a tendency to establish group hierarchy and compete with members of an out-group. The subscale contains four items (e.g., “I usually sacrifice my self-interest for the benefit of my group”). Participants responded on a scale from 1 (*strongly disagree*) to 5 (*strongly agree*) and the mean scores were calculated. Confirmatory factor analysis^1^ showed that the four-factor model exhibited acceptable model fit (χ^2^(71) = 178.3, *p* < 0.001, CFI = 0.94, TLI = 0.92, RMSEA = 0.055).

*Consideration of Future Consequences* is a 14-item scale [43] that measures the extent to which people consider either distant outcomes (CFC-Future) or immediate outcomes of their behaviour (CFC-Immediate). For the current study we used a Slovak translation [44]. The scale has 14 items forming two factors: CFC-Future (e.g., “When I make a decision, I think about how it might affect me in the future.”) and CFC-Immediate (e.g., “I only act to satisfy immediate concerns, figuring the future will take care of itself”). Participants respond on a scale from 1 (*strongly disagree*) to 7 (*strongly agree*) and the mean scores were calculated. Two-factor model showed good fit in CFA (χ^2^(76) = 174.9, *p* < 0.001, CFI = 0.97, TLI = 0.96, RMSEA = 0.051).

*Prosocial tendencies* were measured by the 23-item Prosocial Tendencies Measure (PTM) [45, 46]. Exemplary items are: “I think that one of the best things about helping others is that it makes me look good” or “I never hesitate to help others when they ask for it”. Participants responded on a scale from 1 (*not at all like me*) to 5 (*absolutely like me*). Because in this study we were interested in general prosocial tendencies—regardless of specific motivations, we calculated a summary score. One-factor model that only included the nine items of the dimensions emotional, dire and compliant was estimated based on the personal communication with one of the original authors of the scale. This model exhibited acceptable fit (χ^2^(27) = 99.6, *p* < 0.001, CFI = 0.97, TLI = 0.96, RMSEA = 0.073).

#### Emotional reactions

*Environmental concerns* were measured by three items related to how threatened participants felt by climatic changes when thinking about health (their own and their loved ones), quality of life (their own and their loved ones), and economic and social consequences adapted from Kohút et al. [47]. Participants answered on a scale from 1 (*completely disagree*) to 7 (*completely agree*) and the mean score was calculated.

*Feelings of helplessness* were measured by four items related to how a participant felt about climatic changes adapted from Šrol et al. [48], e.g., “I feel helpless when thinking about the present situation caused by climate change”. Participants answered on a scale from 1 (*completely disagree*) to 7 (*completely agree*) and the mean score was calculated.

Two-factor model with concern and helplessness factors loaded by their three and four items, respectively, showed very good model fit in CFA (χ^2^(13) = 15.5, *p* = 0.277, CFI = 0.99, TLI = 0.99, RMSEA = 0.020).

#### Outcome variable

*Pro-environmental behaviour* (PEB) was measured by eleven self-reported items related to the behaviour of participants regarding recycling and waste avoidance (4 items), purchasing behaviour (4 items), and energy conservation (3 items). Participants chose their answers from three options and we assigned 1 point for answers indicating most environmentally harmful answers, 2 points for answers indicating some pro-environmental action/willingness and 3 points for answers indicating deliberate effort put into PEB. The mean score was used. A CFA model with one factor showed good fit to the data (χ^2^(44) = 70.2, *p* = 0.007, CFI = 0.97, TLI = 0.97, RMSEA = 0.035).

## Results

As the first step in our analyses, we examined the correlations between PEB, environmental concern, helplessness, and outward orientation. Given that we measured multiple potentially associated dimensions of outward orientation, our next step was to examine whether these dimensions can be reduced to a smaller number of meaningful outward orientation components using the principal components analysis. Finally, using hierarchical linear regression, we examined whether outward orientation, environmental concern, and helplessness predict PEB over and above demographic factors and tested for potential moderating effects of helplessness and outward orientation in the relationship between environmental concern and PEB.

### Correlations between main variables

Pearson’s correlations between all variables reported in the present study are presented in Table 3. As can be seen form the table, PEB is moderately positively correlated with environmental concern, prosocial tendencies, future orientation, and horizontal collectivism. The correlations with helplessness and individualism are also positive although they are very weak. The only aspect of outward orientation that was negatively correlated (weakly) with PEB was immediate orientation. Overall, demographic variables did not show substantial associations with other variables in the study with several exceptions. Older people reported slightly more PEB, as well as more prosocial tendencies, higher collectivism, and lower vertical individualism. Women reported higher levels of emotional reactions (concern and helplessness) and prosociality but lower vertical individualism. And people with higher education considered future consequences slightly more (and immediate consequences slightly less) and reported higher vertical collectivism.

**Table 3:** Pearson’s correlations between all variables in the present study

	1	2	3	4	5	6	7	8	9	10	11	12
1. Pro-environmental behaviour	1											
2. Environmental concern	**0.26**	1										
3. Helplessness	**0.13**	**0.58**	1									
4. Prosocial tendencies	**0.19**	**0.24**	**0.17**	1								
5. Future consequences	**0.28**	**0.37**	**0.22**	**0.48**	1							
6. Immediate consequences	**− 0.13**	− 0.02	**0.10**	0.01	**− 0.14**	1						
7. Horizontal collectivism	**0.25**	**0.20**	**0.10**	**0.51**	**0.37**	0.01	1					
8. Vertical collectivism	0.06	**0.24**	**0.16**	**0.41**	**0.31**	0.05	**0.56**	1				
9. Vertical individualism	**0.12**	**0.11**	0.05	**0.12**	**0.20**	**0.14**	**0.29**	**0.28**	1			
10. Horizontal individualism	**0.09**	0.00	0.05	**0.13**	**0.24**	**0.10**	**0.12**	0.05	**0.19**	1		
11. Age	**0.12**	− 0.02	− 0.08	**0.14**	− 0.07	− .0.02	**0.11**	**0.20**	**0.09**	**− 0.19**	1	
12. Gender (1 = men, 2 = women)	− 0.00	**0.11**	**0.10**	**0.12**	0.08	− 0.01	0.07	0.06	**− 0.21**	0.04	**− 0.09**	1
13. Education	0.06	− 0.00	0.02	− 0.05	**0.14**	**− 0.11**	0.04	**0.11**	0.03	0.01	0.04	− 0.05

Correlations are based on 500 observations (those with education are based on 497 observations due to three missing values). Significant correlations (*p* < 0.05) are presented in bold. Correlations with absolute value of *r* > 0.088 are significant at *p* < 0.05, values of *r* > 0.116 are significant at *p* < 0.01, and correlations of *r* > .0.147 are significant at *p* < 0.001

Finally, some of the aspects of outward orientation, such as prosociality, future orientation and collectivism traits, were substantially mutually associated, while others shared virtually no correlations. Specifically, prosociality was strongly correlated with future orientation (*r* = 0.48), as well as horizontal (*r* = 0.51) and vertical collectivism (*r* = 0.41), but shared no correlation with immediate orientation and only weak correlations with individualism (*r*’s < 0.13). This could indicate the former variables (prosociality, future orientation, and collectivism) may reflect some common aspect of outward orientation (see Sect. "Analysis of components of outward orientation" below).

### Analysis of components of outward orientation

To address the mutual interrelationships among the different aspects of outward orientation, we examined whether these dimensions can be reduced to a smaller number of meaningful components. To this end, we have conducted a principal component analysis with varimax rotation.^2^ Based on the parallel analysis, two components were identified, which cumulatively explained 53% of variance in the indicators (for more detailed information about the principal component analysis, see Section A of the Additional file 1. The first component was highly loaded with prosociality (λ = 0.78), horizontal collectivism (λ = 0.78), vertical collectivism (λ = 0.70), and future orientation (λ = 0.73). The second component, on the other hand, consisted of immediate orientation (λ = 0.77), vertical individualism (λ = 0.62), and horizontal individualism (λ = 0.50). The two components—representing outward and self-centred orientation, respectively—were retained and used in the analyses below. The analysis of associations between outward and self-centred orientation and individual PEB measured in the present study can be found in Section B of the Additional file 1.

### Predicting pro-environmental behaviour

As the next step in our analysis, we wanted to see whether environmental concern, helplessness, and outward orientation predict PEB individually and above demographic factors. To this end, we conducted a hierarchical linear regression where gender, age, and education were entered in the first step, and the second step included emotional reactions and outward orientation components. As can be seen in Table 4, in the final model, the predictors explained approximately 11% of the variance in PEB. This was mostly due to environmental concern and outward orientation component which both predicted PEB to a similar extent. After accounting for other predictors in the model, both helplessness and the self-centred orientation component showed negative, but negligible regression coefficients. However, among the demographic variables, age showed up to be weakly but positively predictive of PEB. Please note that we have also repeated this regression using a structural equation modelling (SEM) approach (see Section D of the Additional file 1) and the results were not substantially different from those presented here (the only categorical difference was that education showed up as a significant positive predictor in the analysis using SEM).

**Table 4:** Summary of the hierarchical linear regression predicting self-reported pro-environmental behaviour

		*β*	*95% CI*	*t*	*p*
*Step 1*		Δ*R*^*2*^ = 0.018, *F*(3,493) = 2.99, *p* = 0.031			
Gender		− 0.04	[− .0.12, 0.05]	− 0.818	0.414
Age		**0.09**	**[0.01, 0.18]**	**2.16**	**0.032**
Education		0.04	[− 0.04, 0.12]	0.913	0.361
*Step 2*		Δ*R*^*2*^ = 0.109, *F*(4,489) = 15.2, *p* < 0.001			
Environmental concern		**0.21**	**[0.10, 0.32]**	**3.86**	** < 0.001**
Helplessness		− 0.02	[− 0.12, 0.08]	− 0.377	0.706
Outward orientation		**0.21**	**[0.12, 0.30]**	**4.57**	** < 0.001**
Self-centred orientation		− 0.03	[− 0.11, 0.06]	− 0.604	0.546
*Full model*		adj. *R*^*2*^ = .0.114, *F*(7,489) = 10.12, *p* < 0.001			

The table shows standardized regression coefficients (*β*), *t*-values with their respective 95% confidence intervals and significance at the final step of the model, as well as the change in model fit at both steps (Δ*R*^*2*^). Significant regression coefficients (*p* < 0.05) are presented in bold0

### Moderating effects of outward orientation and helplessness

Finally, to address the possibility of closing the green gap, we examine the potential moderating effects of outward orientation and helplessness on the association between environmental concern and PEB. To this end, we used the regression model presented in Table 4 above and added interaction terms between environmental concern, helplessness, and outward orientation in the third step of the regression to see whether these predictors explain any additional variance in PEB (see Section C in the Additional file 1 for the full summary of the two regression models).

**Fig. 1:**
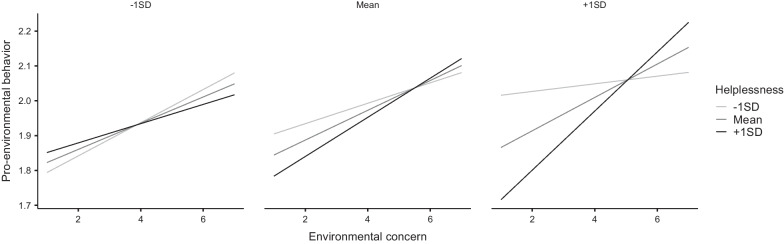
Estimated marginal means plot from the linear regression showing the interaction between environmental concern, helplessness, and outward orientation as predictors of pro-environmental behaviours. The three panels depict predictors at 1 SD below mean (left panel), mean (centre), and 1 SD above mean (right) in outward orientation

Adding the four possible interaction terms between concern, helplessness, and outward orientation to the regression model led to a significant improvement in the fit of the model, Δ*R*^*2*^ = 0.030, *F*(4,485) = 4.29, *p* = 0.002, with the three-way interaction term emerging as a significant predictor in the model (β = 0.11, 95% CI [0.05, 0.17], *p* < 0.001). To decompose this moderating effect, we first present estimated marginal means plots showing how environmental concern predicts PEB on low, average, and high levels (mean and one standard deviation above and below mean) of helplessness and outward orientation (see Fig. 1). Secondly, using the SPSS Process macro by Hayes [42], we generated some additional output to better understand the moderated moderation effect. Specifically, as can be seen from the right panel of Fig. 1., there was a two-way interaction between concern and helplessness in predicting PEB, but this was only significant among people with high (+ 1 SD above mean) outward orientation, *F*(1,485) = 15.19, *p* < 0.001 and those with average, *F*(1,485) = 4.12, *p* = 0.043, but not low outward orientation, *F*(1,485) = 0.405, *p* = 0.525. Also, as is visible from Fig. 1, environmental concern predicted PEB in all participants except those with simultaneously low levels of helplessness and high levels of outward orientation and those with high levels of helplessness and low levels of outward orientation.

**Fig. 2:**
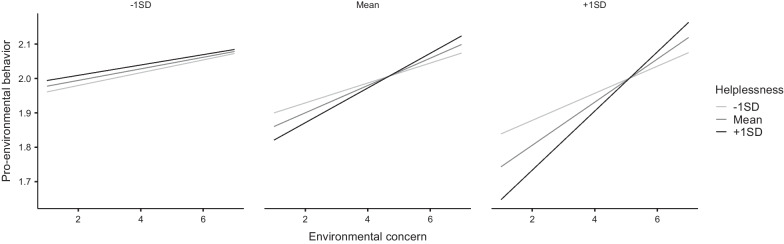
Estimated marginal means plot from the linear regression showing the interaction between environmental concern, helplessness, and self-centred orientation as predictors of pro-environmental behaviours. The three panels depict predictors at 1 SD below mean (left panel), mean (centre), and 1 SD above mean (right) in self-centred orientation

Exploring the potential interactions of predictors with the self-centred orientation showed that adding the interaction terms between concern, helplessness, and self-centred component in the regression model presented in Table 4 likewise led to a significant increase in explained variance, Δ*R*^*2*^ = 0.024, *F*(4,485) = 3.40, *p* = 0.009, with the three-way interaction term emerging as a significant predictor in the model (β = 0.07, 95% CI [0.01, 0.12], *p* = 0.022). Importantly, again there was a two-way interaction between concern and helplessness (see right panel of Fig. 2) in predicting PEB, but this was only significant among those with high levels of self-centred orientation, *F*(1,485) = 7.20, *p* = 0.008, not average, *F*(1,485) = 2.64, *p* = 0.105, or low levels thereof, *F*(1,485) = 0.02, *p* = 0.890. Environmental concern was a significant predictor of p PEB in all participants except those with low levels of self-centred orientation, regardless of their helplessness levels.

## Discussion

Driven by the study by Tam and Chan [3], the present paper aimed to explore the potential of individual characteristics jointly referred to as outward orientation in closing the green gap. Being aware of the role of emotional responses, we also investigated the moderating role of helplessness in the relation between environmental concerns and PEB. The study contribution is twofold. First, it indicates that individual characteristics and values jointly referred to as outward/self-centred orientation could explain PEB above demographic factors. More importantly, our results show that those characteristics could serve as an antidote for heightened helplessness. Second, the study also contributes to the theory by discussing the role of individual characteristics and values in stimulating PEB and, thus, closing the green gap.

### The relation between individual characteristics, values, environmental concerns and PEB

In line with the literature, our results show a positive, but not a very strong correlation between environmental concerns and PEB, corroborating the existence of the green gap [2, 3, 7]. Surprisingly, helplessness was also positively, albeit weakly, correlated with PEB and strongly with environmental concerns. Unlike in a recent study by Pickering and Dale [50], given their heightened environmental concern, helpless individuals seem to be relatively passive. The findings are consistent with the extant literature and international surveys showing that helplessness predominates among feelings related to climate change [1, 38]. Consequently, it may deprive individuals of the sense of agency and, thus, set bounds on their involvement in PEB.

Furthermore, in accordance with the expectations, horizontal collectivism, future orientation, and prosociality—as components of the outward orientation construct—showed to be positively related to PEB and environmental concern but also helplessness. In line with Landry et al. [7], this could indicate that emotional responses to the environmental crisis—in this study proxied by concerns and helplessness—could share some joint antecedents such as a general tendency to worry that results in environmental disengagement [51, 52]. Future research is needed to pinpoint this confounding variable or to identify measures increasing environmental self-efficacy without hampering the sense of concern as it could be one of the crucial aspects in closing the green gap [53]. Interestingly, the only variable that showed a negative (albeit weak) correlation with PEB was immediate orientation and no variable was significantly negatively related to environmental concerns. Our findings indicate that despite the existence of the green gap, both environmental concerns and at least some PEB (e.g., waste separation, passive energy saving) are widespread in society (for more information, see Additional file 1). It seems, thus, that when it comes to environmental concerns and PEB it is no longer a question of *whether* people are involved but rather *to what extent*.

Generally, the data show a straightforward pattern related to outward orientation: the more concerned and the more outwardly oriented individuals are, the more PEB they adopt. The only exception included highly outwarded individuals with low helplessness for whom concerns did not predict PEB. This corroborates the view that outward orientation could counterbalance negative emotional responses which could result in environmental idleness. As the literature about value orientations shows, individuals who see themselves as a part of the global community, are willing to transcend their selfish interests and have more egalitarian views about the world may adopt more PEB [14, 16, 17, 25]. The results show similar patterns to those observed by Loy and Spence [54] showing that heightened outward orientation may play a role in reducing the spatial, social, and temporal distance to the consequences of climate change and, thus, fostering the sense that environmental issues matter for all people.

For self-centred individuals, in turn, we observed a consistent drop in PEB with the increase in self-centred orientation. The more helpless they felt, the more visible the drop was. However, when they reported their concerns to be high, their self-centred orientation ceased to be an obstacle in performing PEB. The data show another interesting pattern: People with the *lowest* self-centred orientation (-1 SD) reported relatively high involvement in PEB regardless of their concerns for climate change or feelings of helplessness. On the other hand, people with the *highest* self-centred orientation (+ 1 SD) also reported more PEB but only when their concern for the climate was high. Thus, increasing concerns among self-centred individuals has a potential to boost their involvement in PEB. Similar patterns were observed in individualistic countries where concerned individuals were willing to pay more to mitigate the negative effects of climate change when they were sufficiently concerned [26]. Given our results, it seems possible that self-centred individuals may be willing to overcome their self-interest with the view to mitigating climate change if they perceive it as a significant threat. Stressing the importance of values, Chan [18] observed that when personal and social values are in conflict, collectivists may more easily subordinate their personal interests to the common good. Individualists, in turn, may resolve the inconsistency between personal and community interests in favour of the former. Therefore, it is important that self-centred individuals internalise environmental values and reconcile them with personal norms [17]. Once environmental values—and concerns—become an important part of individualists’ self-concept, they will be more likely to adjust their actions to avoid the discrepancy between values and behaviour. Otherwise, they are likely to free-ride on others’ efforts even if they sense environmental concerns [55].

### Individual characteristics and types of PEB

The results could be driven by the type of PEB employed in the present study and the fact that various PEB may appeal to different individuals [10, 55, 56]. We have observed that certain PEB, such as waste sorting, some level of energy-saving or avoiding buying clothes one does not need, are frequently reported by both outwardly and self-centred oriented people. Specifically, recycling and waste avoidance were common while zero waste behaviour, upcycling clothes and vegetarian/vegan nutrition preferences were among the least popular PEB regardless of orientation. The most distinct differences between highly self-centred and outwardly oriented individuals were observed in energy conservation. Although highly self-centred individuals reported buying efficient bulbs, this was not followed by more active commitment (e.g., turning off the light). Energy conservation allows for considerable savings in addition to any environmental benefits it brings but self-centred people seem to wish to achieve the savings with minimum effort. They may not be driven by prosocial or outward tendencies but by self-interested motivations instead. Nevertheless, it seems that self-centred individuals may still be attracted by PEB if either they internalise environmental concern or sufficient incentives are put in place. Once self-centred individuals recognise that benefits of PEB outweigh costs (by financial savings, boosting their ego, building reputation or manifesting status), they may remain self-interested and yet behave sustainably [10, 57, 58]. Morally, we could praise selfless acts. From the pragmatic perspective, however, what matters is the environmental effect. If sustainability for some individuals is merely a side effect of their self-centred motivations, then so be it.

### Theoretical implications—introducing outward orientation

The study also provides first and preliminary results corroborating the view that individual characteristics and values could explain PEB better than demographic characteristics. In our analysis, four variables—horizontal and vertical collectivism, future orientation and prosocial motivations—showed up to be interrelated, and could be meaningfully combined into a single component capturing outward, selfless tendencies. Three other variables—horizontal and vertical individualism and immediate orientation—represented an orthogonal component of self-centred orientation that negatively moderated the relations with sustainable behaviour. Thus, the results extend the current literature about value orientations by showing that other characteristics indicating that an individual is concerned about wellbeing of other people (including future generations) could inform the debate about bridging the green gap. Whether the constructs have the potential to explain sustainable behaviour above and beyond other psychological constructs, including personality, requires further and thorough investigation. Nevertheless, it seems that outward orientation could provide a promising avenue for future research on PEB and the green gap. Future research could explore the validity of these preliminary components and their relations with other forms of pro-environmental and, more broadly, prosocial behaviour.

### Practical implications for communication strategies

Our results show that although moderate involvement in PEB is relatively widespread, there is still considerable potential for improvement. Policy-wise, this indicates that there is a need for enhancing more universal commitment and removing barriers to adopting advanced or burdensome PEB (e.g. zero waste behaviour, upcycling clothes and low meat or meat-free diet). The data show, that particularly, the promotion of plant-based eating habits is a domain with great potential to progress (see Additional file 1). Yet, to gain support, the policies need to be aligned (or at least not conflicted) with values endorsed by individuals [10, 57]. Thus, universal strategies are unlikely to be effective in building widespread support for PEB. Instead, communications should be more tailored and targeted separately at individuals with either outward or self-centred orientation addressing their potentially differential motivations [58]. The data allow us to identify at least two communication strategies that should be applied in parallel to elicit widespread behavioural responses.

The first strand of communication should be directed at individuals sharing values associated with outward orientation. As the data show, for outwardly oriented individuals, involvement in PEB increases with environmental concern. Therefore, apart from raising concerns communications need to indicate that it is our shared responsibility to mitigate climate change. By this, such communications are likely to appeal to individuals holding outward values dear and understanding that PEB manifest their responsibility for the entire community and future generations. Second, the results show that self-centred individuals are less committed to PEB unless they are highly concerned and may focus on personal rather than economic or social gains associated with PEB (e.g. savings linked with energy conservation, signalling status by buying ostensibly green products). Consequently, to raise environmental concerns and help to internalise them by self-centred individuals, communication strategies should picture environmental threats as real and close even in currently relatively safe regions such as Slovakia. The second important aspect of such communications would be to stress possible personal gains of PEB ranging from economic savings, through ego boosts and alleviating negative emotional responses associated with the concerns, to health benefits. Finally, it is necessary to a sustain balance between the two strategies as the prevalence of one type may backfire against individuals who endorse conflicting values and may be discouraged by incongruent communications [56].

### Limitations and future directions

Although the present study involved a large, representative sample, it is cross-sectional and, thus, the results need to be interpreted with caution. First of all, it is possible that people with different personal characteristics could be prone to response bias to various extents—for instance, due to elevated image concerns or to avoid cognitive dissonance. Future research could focus on an observational or experimental approach to capture actual behaviour and its drivers to provide bias-free insights into the involvement in PEB among various groups of individuals. However, given the fact that most data on the green gap come also from self-reports, our results seem to provide a considerable approximation of the widely investigated phenomenon. Also, future research could investigate the robustness of the findings related to outward and self-centred orientations across various cultural contexts. Specifically, to investigate the effects in cultures that are considered either strongly individualistic or collectivistic. Second, our study focused on a specific subset of private PEB. Future research could extent the catalogue to more diverse PEB such as activism.

Third, it should be noted that while the moderation analyses presented in our study showed that it is important to consider how outward orientation, helplessness, and environmental concern interact in predicting PEB, from the standpoint of practical significance, the identified moderating effects were relatively small (as showed by the increase in *R*^2^). Still, we believe that these effects should not be disregarded as they point out important boundary conditions that help us to understand how to motivate different individuals with particular characteristics and different levels of emotional reactions to act more pro-environmentally. As we already know from such efforts, one size does not fit all. Therefore, such more fine-grained analyses could offer important new information in designing interventions aimed at increasing PEB. Finally, the present study did not investigate barriers—other than helplessness—that hamper changing environmental concerns into sustainable behaviour. It seems likely that depending on individual characteristics, people could perceive different barriers as crucial obstacles in adopting more PEB. Together with different motivations, recognising that the barriers are perceived differently could help to formulate more efficient and targeted policies and interventions encouraging wider groups of individuals to behave sustainably.

## Conclusions

Our results show that policy-makers and environmental organisations need to acknowledge a considerable diversity among agents. The diversity could result in various people being driven by different motivations. Nevertheless, it emerges from our results that the involvement in PEB may be easier to achieve among individuals with strong outward orientation. It is sufficient to arouse concerns among them and nurture their sense of relatedness both with the global community and future generations. However, a comforting message that could be derived from the current study is that strongly self-centred individuals are not immune to environmental concerns. Once they experience high concerns over the environment, they tend to act equally eagerly as outwardly oriented individuals. Policy-wise, that could indicate at least two paths of increasing global involvement in PEB and bridging the green gap parallel to increasing environmental concerns: boosting the sense of social responsibility among those prone to outward orientation and stressing individual benefits for those who are self-centred. Importantly, both those paths potentially outweigh helplessness known to be a considerable drawback in fostering a more active approach to mitigating climate change. Instead of forcing people into frames that do not fit their values, policy-makers should provide various opportunities to reap the benefits—psychological, social or economic—of environmentally concerned behaviour for those whose scope may seem too narrow or myopic.

## Supplementary Information

**Additional file 1.** Suplementary material.

## Data Availability

Raw data and supplementary materials are available at: https://osf.io/dptzw. In case of question or data and materials request, please contact Magdalena Adamus (magdalena.adamus@savba.sk).

## Abbreviations

CFC: Considerations of future consequences
ESS: European Social Survey
HC: Horizontal collectivism (HVIC subscale)
HVIC: Horizontal/vertical individualism/collectivism scale
PEB: Pro-environmental behaviour
PTM: Prosocial tendencies measure
VC: Vertical collectivism (HVIC subscale)

## Footnotes

1 All CFA analyses were conducted in *R* package lavaan [57] and estimated using DWLS.

2 Originally, we ran the principal component analysis with oblimin rotation—thus, allowing the components to be correlated. However, the resulting components shared a negligible correlation (*r* = .072). Therefore, we redid the analysis with the varimax rotation, thus leaving us with two uncorrelated components.
