# Supplementary material for: Seeing past the tip of your own nose? How outward and self-centred orientations could contribute to closing the green gap despite helplessness
Source: BMC Psychol. 2023 Mar 24;11:79. doi: 10.1186/s40359-023-01128-z (PMC10037357; doi:10.1186/s40359-023-01128-z)
Supplement: Supplementary file 1 — Additional file 1. Suplementary material. [file 40359_2023_1128_MOESM1_ESM.docx]

**Supplementary Material**

**SECTION A**

**Principal component analysis of outward orientation indicators**

In section 3.2 of the main manuscript, we describe a principal component analysis that was used to examine whether the dimensions of outward orientation in our study can be reduced to a smaller number of meaningful components of outward orientation. To this end, we run a principal component analysis with varimax rotation in jamovi software. The assumptions for the principal component analysis were met as indicated by the significant Bartlett’s test, χ^2^(21) = 641, *p* < .001, and KMO measure of sampling adequacy (overall KMO = .714). Based on the parallel analysis (see Fig. S1), we identified two components with eigenvalues of 2.53 and 1.18 and 36.1% and 16.9% of explained variance, respectively. Component loadings can be found in Table S1 below.

**
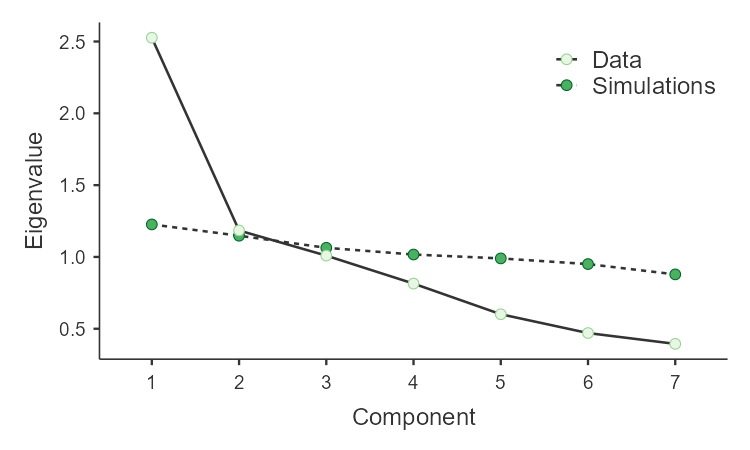
**

**Figure S1**. Parallel analysis for the principal component analysis with the seven indicators of outward orientation (prosociality, future and immediate orientation, two collectivism and two individualism indicators)

| **Table S1**. Summary of the principal component analysis | | | |
| --- | --- | --- | --- |
|  | Component | |  |
|  | 1 | 1 | Uniqueness |
| Hierarchical collectivism | .78 |  | .357 |
| Vertical collectivism | .70 |  | .464 |
| Vertical individualism |  | .62 | .526 |
| Hierarchical individualism |  | .50 | .719 |
| Prosociality | .78 |  | .397 |
| Future orientation | .73 |  | .465 |
| Immediate orientation |  | .77 | .361 |
| *Note.* The table shows component loadings for the two component identified via parallel analysis and uniqueness for every indicator. The components are based on the varimax rotation. For the ease of interpretation, component loadings below .30 are suppressed from the table. In the text of the main article, the first component is labelled “outward orientation” and the second component “self-centred orientation”. | | | |

**SECTION B**

**Associations between individual pro-environmental behaviors and outward and self-centred orientation**

To test for the association between outward and self-centred orientation and individual pro-environmental behaviors (PEBs) measured in our study, we conducted a series of chi-square tests. In every test, outward and self-centred orientation were treated as two grouping variables created by a median split of the continuous component variables used in the regressions in the main manuscript. Both of these grouping variables were then compared against every one of the individual pro-environmental behaviors measured in our study via a chi-squared test, with individual column proportions further compared by a z-test. The results for eleven surveyed behaviors are shown in Figs. S2–S12.

In general, outward orientation showed association with almost every surveyed PEB, with the exception of buying clothes and shoes, eating animal products, saving energy and efficient heating. Self-centred orientation, on the other hand, was only significantly associated with considering the origin of clothes, reduced use of single use plastics and saving energy as forms of PEB. Also, notably, some PEBs were relatively frequent, meaning that even participants with high self-centred and low outward orientation mostly reported engaging in them to moderate or high levels. For example, only 3.6% of the sample reported not sorting any waste, 4.4% reported not doing anything to save energy, and 7.6% reported buying clothes and shoes once a month regardless of whether they need them, and there were no differences in these response options with regard to the either outward or self-centred orientation. On the other hand, some behaviors were relatively infrequent with very few people reporting engaging in those behaviors to the highest level. Among these were being vegetarian or vegan (2.6%), using mostly upcycled clothes (4.2%), zero waste living (10.8%), and buying clothes or shoes when the old ones cannot be used anymore (12.4%). Although there were some differences in these response options between people with low and high outward orientation, these differences were relatively small and generally, these behaviors were quite infrequently employed by our participants regardless of their orientation.


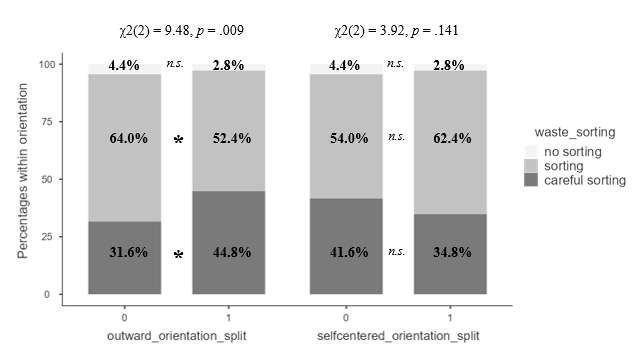


**Figure S2**. Stacked bar plot showing the results of chi-squared test of association between waste sorting and outward (left panel) and self-centred orientation (right panel). The bars show percentages of waste sorting response options within the columns. Column proportions are compared via z-test, significant differences (*p* < .05) are marked with asterisk (*).


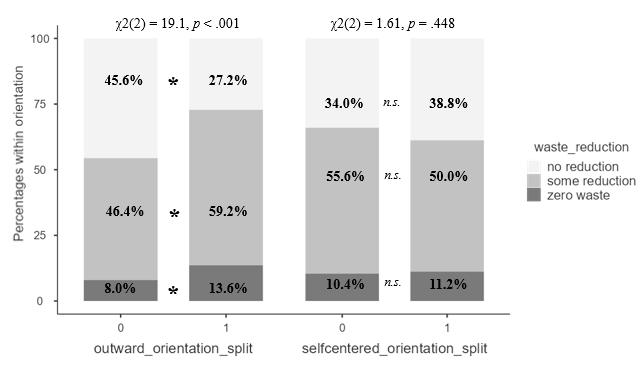


**Figure S3**. Stacked bar plot showing the results of chi-squared test of association between waste reduction and outward (left panel) and self-centred orientation (right panel). The bars show percentages of waste reduction response options within the columns. Column proportions are compared via z-test, significant differences (*p* < .05) are marked with asterisk (*).


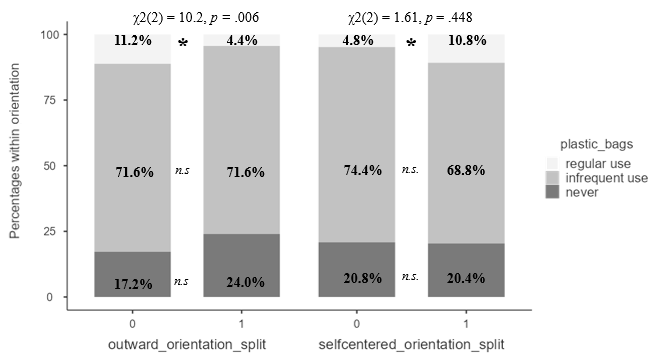


**Figure S4**. Stacked bar plot showing the results of chi-squared test of association between the use of plastic bags and outward (left panel) and self-centred orientation (right panel). The bars show percentages of plastic bag use response options within the columns. Column proportions are compared via z-test, significant differences (*p* < .05) are marked with asterisk (*).


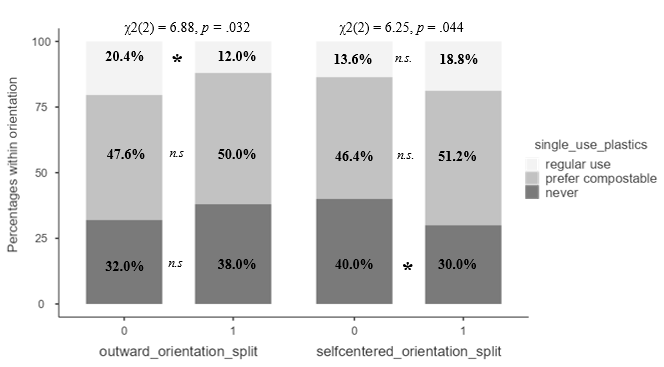


**Figure S5**. Stacked bar plot showing the results of chi-squared test of association between the single-use plastics use and outward (left panel) and self-centred orientation (right panel). The bars show percentages of single-use plastics use response options within the columns. Column proportions are compared via z-test, significant differences (*p* < .05) are marked with asterisk (*).


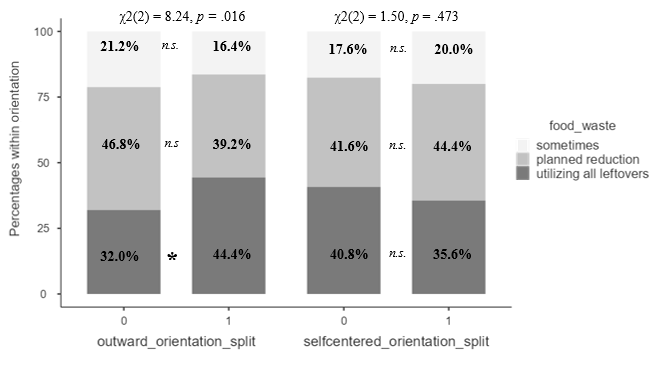


**Figure S6**. Stacked bar plot showing the results of chi-squared test of association between the food waste and outward (left panel) and self-centred orientation (right panel). The bars show percentages of food waste response options within the columns. Column proportions are compared via z-test, significant differences (*p* < .05) are marked with asterisk (*).


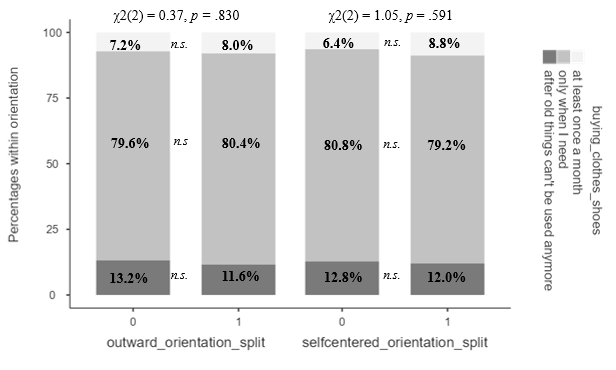


**Figure S7**. Stacked bar plot showing the results of chi-squared test of association between buying clothes and shoes and outward (left panel) and self-centred orientation (right panel). The bars show percentages of buying clothes and shoes response options within the columns. Column proportions are compared via z-test, significant differences (*p* < .05) are marked with asterisk (*).


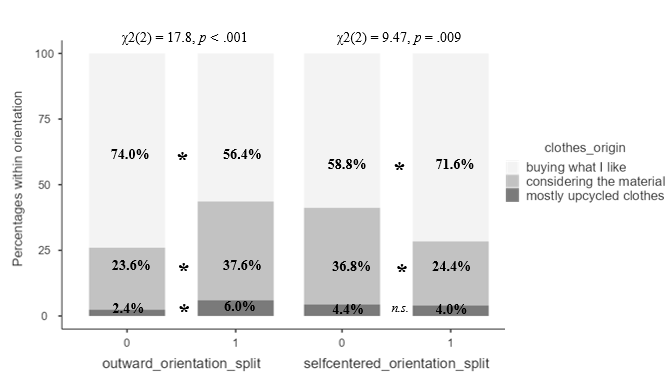


**Figure S8**. Stacked bar plot showing the results of chi-squared test of association between considering clothes origin and outward (left panel) and self-centred orientation (right panel). The bars show percentages of considering clothes origin response options within the columns. Column proportions are compared via z-test, significant differences (*p* < .05) are marked with asterisk (*).


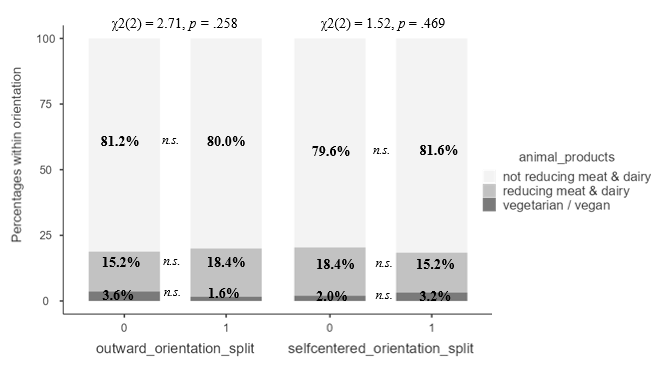


**Figure S9.** Stacked bar plot showing the results of chi-squared test of association between eating animal products and outward (left panel) and self-centred orientation (right panel). The bars show percentages of eating animal products response options within the columns. Column proportions are compared via z-test, significant differences (*p* < .05) are marked with asterisk (*).


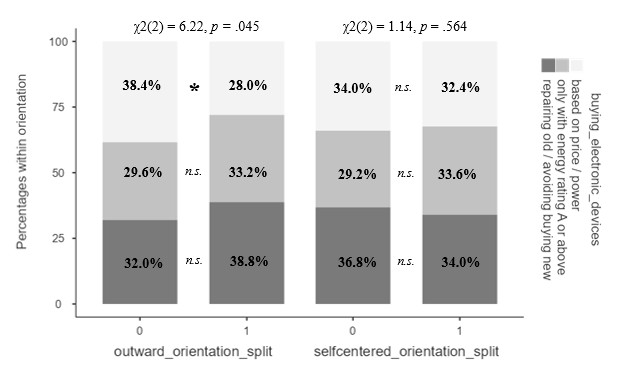


**Figure S10**. Stacked bar plot showing the results of chi-squared test of association between buying electronic devices and outward (left panel) and self-centred orientation (right panel). The bars show percentages of buying electronic devices response options within the columns. Column proportions are compared via z-test, significant differences (*p* < .05) are marked with asterisk (*).


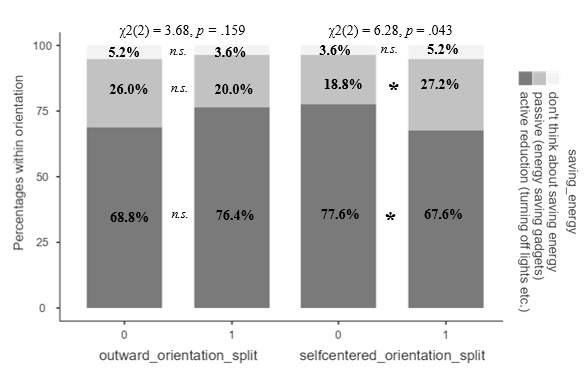


**Figure S11**. Stacked bar plot showing the results of chi-squared test of association between saving energy and outward (left panel) and self-centred orientation (right panel). The bars show percentages of saving energy response options within the columns. Column proportions are compared via z-test, significant differences (*p* < .05) are marked with asterisk (*).


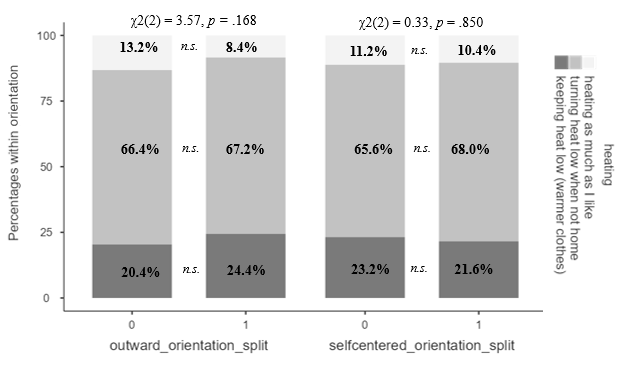


**Figure S12**. Stacked bar plot showing the results of chi-squared test of association between heating and outward orientation (left panel) and self-centred orientation (right panel). The bars show percentages of heating response options within the columns. Column proportions are compared via z-test, significant differences (*p* < .05) are marked with asterisk (*).

**SECTION C**

**Regression models examining potential moderating effects of outward / self-centred orientation and helplessness on the association between environmental concern and pro-environmental behavior**

In the main manuscript, we only present the results of a three-way interaction between outward / self-centred orientation, helplessness and concern as a predictor of pro-environmental behavior and decompose this interaction. Below, in Table S2 and S3, we include the full summaries of two regressions with added interaction terms.

| **Table S2**. Summary of the hierarchical linear regression predicting self-reported pro-environmental behaviour with interacting effects of outward orientation, helplessness and environmental concern | | | | |
| --- | --- | --- | --- | --- |
|  |  | *β* | *t* | *p* |
| *Step 1* | | Δ*R^2^* = .018, *F*(3,493) = 2.99, *p* = .031 | | |
| Gender | | –.04 | –0.866 | .387 |
| Age | | **.10** | **2.45** | **.015** |
| Education | | .03 | 0.658 | .511 |
|  | |  |  |  |
| *Step 2* | | Δ*R^2^* = .109, *F*(4,489) = 15.2, *p* < .001 | | |
| Environmental concern | | **.23** | **4.21** | **<.001** |
| Helplessness | | –.05 | –0.906 | .365 |
| Outward orientation | | **.12** | **2.485** | **.013** |
| Self-centred orientation | | –.04 | –0.978 | .329 |
|  | |  |  |  |
| *Step 3* | | Δ*R^2^* = .030, *F*(4,485) = 4.29, *p* = .002 | | |
| Environmental concern * helplessness | | **.08** | **2.03** | **.043** |
| Outward orientation * helplessness | | –.02 | –0.522 | .602 |
| Outward orientation * environmental concern | | .03 | 0.585 | .559 |
| Outward orientation * environmental concern * helplessness | | **.11** | **3.38** | **<.001** |
| *Full model* | | adj. *R^2^* = .137, *F*(11,485) = 8.17, *p* < .001 | | |
| *Note.* The table shows standardized regression coefficients (*β*), *t*-values and their significance at the final step of the model, as well as the change in model fit at three steps (Δ*R^2^*). Significant regression coefficients (*p* < .05) are presented in bold. | | | | |

| **Table S3**. Summary of the hierarchical linear regression predicting self-reported pro-environmental behaviour with interacting effects of self-centred orientation, helplessness and environmental concern | | | | |
| --- | --- | --- | --- | --- |
|  |  | *β* | *t* | *p* |
| *Step 1* | | Δ*R^2^* = .018, *F*(3,493) = 2.99, *p* = .031 | | |
| Gender | | –.03 | –0.748 | .455 |
| Age | | **.10** | **2.22** | **.027** |
| Education | | .04 | 0.932 | .352 |
|  | |  |  |  |
| *Step 2* | | Δ*R^2^* = .109, *F*(4,489) = 15.2, *p* < .001 | | |
| Environmental concern | | **.21** | **3.73** | **<.001** |
| Helplessness | | –.01 | –0.167 | .866 |
| Outward orientation | | **.19** | **4.01** | **.013** |
| Self-centred orientation | | **–.11** | **–2.02** | **.044** |
|  | |  |  |  |
| *Step 3* | | Δ*R^2^* = .024, *F*(4,485) = 3.40, *p* = .009 | | |
| Environmental concern * helplessness | | .06 | 1.63 | .105 |
| Self-centred orientation * helplessness | | –.04 | –0.804 | .422 |
| Self-centred orientation * environmental concern | | **.12** | **2.25** | **.025** |
| Self-centred orientation * environmental concern * helplessness | | **.07** | **2.29** | **.022** |
| *Full model* | | adj. *R^2^* = .131, *F*(11,485) = 7.80, *p* < .001 | | |
| *Note.* The table shows standardized regression coefficients (*β*), *t*-values and their significance at the final step of the model, as well as the change in model fit at three steps (Δ*R^2^*). Significant regression coefficients (*p* < .05) are presented in bold. | | | | |

**SECTION D**

**Regression in Section 3.3 using structural equation modelling (SEM)**

In the main manuscript, we present the results of an ordinary least squares regression predicting pro-environmental behavior with outcome variable and predictors included as observed variables (outward orientation variables are included as component scores). Here, we recreate the same regression with the structural equation modelling approach using *R* package lavaan. First, we estimated the measurement model with DWLS estimator for a five-factor latent model. Factors helplessness, environmental concern and pro-environmental behavior were loaded by their individual indicators. Two additional factors representing outward orientation variables were loaded by the same indicators as in the principal component analysis described in Section 3.2 of the main manuscript. The measurement model showed good fit to the data (χ^2^(265) = 425.1, *p* <.001, CFI = .97, TLI = .97, RMSEA = .035). Next, we performed a regression with these latent variables predicting pro-environmental behaviour with emotional reactions, outward orientation variables, and demographic variables as covariates. The results are summarized in Table S4. Together, these predictors explained 20% of variance in the pro-environmental behaviour. Overall, these results are consistent with those presented in the main manuscript with the exception of education which showed up as a significant predictor here, but not in the analysis presented in the main manuscript.

| **Table S4**. Summary of the regression predicting self-reported pro-environmental behaviour using structural equation modelling | | | | |  |
| --- | --- | --- | --- | --- | --- |
|  |  | *β* | *z* | *p* | |
| Gender | | .02 | 0.594 | .553 | |
| Age | | **.12** | **3.75** | **<.001** | |
| Education | | **.09** | **2.84** | **.005** | |
| Environmental concern | | **.26** | **4.59** | **<.001** | |
| Helplessness | | –.05 | –1.06 | .289 | |
| Outward orientation | | **.23** | **3.09** | **.002** | |
| Self-centred orientation | | .08 | 0.915 | .360 | |
| *Note.* The table shows standardized regression coefficients (*β*), z-values and significance of every predictor in the regression. Significant regression coefficients (*p* < .05) are presented in bold. | | | | |  |
